# The prognostic value of including S-1 regimens in stage II and III gastric cancer patients: A propensity score matching and subgroup analyses

**DOI:** 10.7150/jca.84535

**Published:** 2023-06-26

**Authors:** Yifan Li, Sujiao Liang

**Affiliations:** 1 Second Department of General Surgery, Shanxi Province Cancer Hospital, Shanxi Hospital Affiliated to Cancer Hospital, Chinese Academy of Medical Sciences, Cancer Hospital Affiliated to Shanxi Medical University, Taiyuan, Shanxi, 030013, Peoples R China.; 2 Department of pharmacy, Shanxi Province Cancer Hospital, Shanxi Hospital Affiliated to Cancer Hospital, Chinese Academy of Medical Sciences, Cancer Hospital Affiliated to Shanxi Medical University, Taiyuan, Shanxi, 030013, Peoples R China.

**Keywords:** gastric cancer, propensity score matching, chemotherapy regimen, S-1

## Abstract

**Background:** Although numerous studies have indicated the increased efficacy of including S-1 in chemotherapy regimens, the effect of S-1 in the treatment of gastric cancer remains to be determined. In this study, we analyzed the prognostic value of chemotherapy regimens of including S-1 for stage II and III gastric cancer patients.

**Methods:** A total of 412 patients with stage II gastric cancer and 902 patients with stage III gastric cancer who received D2 gastrectomy plus adjuvant chemotherapy or neoadjuvant chemotherapy were included in this study. Analysis and comparison at a ratio of 1:1 was performed to reduce the baseline differences. Progression-free survival, overall survival, and recurrence were the main outcome indicators.

**Results:** After propensity score matching (PSM), we found that including S-1 in the chemotherapy regimen was only better than without S-1 in 5-year overall survival (OS) (83.6% VS 68.8%, Log-Rank P=0.005) and 5-year progression free survival (PFS) (71.6% VS 61.5%, Log-Rank P=0.005) for stage II gastric cancer patients. The difference in the recurrence (P=0.102), local-regional recurrence (P=0.062), and distant metastases (P=0.328) between the two groups were not significant. As for the stage III gastric cancer patients, Kaplan-Meier survival curves revealed that including S-1 was inferior to excluding S-1 in OS (P=0.023), but not in PFS(P=0.740). However, the difference in recurrence (P<0.001), local-regional recurrence (P=0.002), and distant metastases (P=0.011) between the two groups were significant. Furthermore, including S-1 increased mortality hazard by 27.2% compared to without S-1 (P=0.023) in the subgroup analyses of OS, but not in the subgroup analyses of PFS (P=0.268).

**Conclusions:** Including S-1 did not exhibit superior effect over excluding S-1 in the prognosis of stage II and III gastric cancer patients, but significantly increased the risk of mortality in stage III gastric cancer patients. Moreover, for patients with stage III gastric cancer, including S-1 significantly increased the recurrence of the disease.

## Introduction

Gastric cancer is the fifth most common cancer and the third leading cause of cancer-related deaths worldwide 1. Although the overall survival (OS) of gastric cancer patients has been improved with the development of D2 gastrectomy 2 and the subsequent adjuvant chemotherapy in recent years, the long-term survival rate is still unsatisfactory 3,4.

S-1 is a fourth-generation oral form of fluoropyrimidine and consists of the 5-fluorouracil prodrug tegafur with two modulators, oteracil and gimeracil 5. Many studies have demonstrated that S-1 is a suitable and tolerable adjuvant chemotherapy for patients with locally advanced gastric cancer who have undergone surgical resection. For example, based on the literature reports, the calculated 1-, 3- and 5-year survival rates are 97.7 % (125/128), 90.6% (116/128), and 88.3% (113/128), respectively, for patients receiving S-1, while they are (126/128), 90.6% (116/128), and 69.5% (89/128), respectively, for patients without S-1. In this study, we explored the survival benefit of including S-1 for patients with stage II and III gastric cancer as well as identified the clinical effects of including S-1 in inhibiting metastasis and recurrence.

## Methods

### Data collection

A total of 412 patients with stage II gastric cancer and 902 patients with stage III gastric cancer underwent gastrectomy for the treatment of gastric cancer from 2002 to 2020 in Shanxi, China, were included in this study. The patient's clinicopathological characteristics including the age of patients when surgery was performed, sex, nerve invasion, vascular invasion, the number of positive lymph nodes, the depth of tumor invasion, number of chemotherapy cycles, TNM stage (according to the 8th edition of the American Joint Board on Cancer), maximum tumor diameter, Lauren classification, retinal metastasis, type of gastrectomy, chemotherapy administration, surgical margin, multi-organ resection, chemotherapy protocol, and the Clavien-Dindo classification, multiple metastases, OS, complications, progression-free survival (PFS), were collected. The number of postoperative chemotherapy cycles, the number of neoadjuvant chemotherapy cycles, medical records, surgical records, and follow-up data were analyzed retrospectively.

The patient inclusion criteria were: (1) Received neoadjuvant chemotherapy or adjuvant chemotherapy before radical gastrectomy; (2) Histologically proven gastric cancer; (3) No major complications after the surgery; (4) With complete clinicopathological and follow-up record; and (5) No other malignancies or causes of death besides gastric cancer.

The patient exclusion criteria were :(1) Without complete clinical record; (2) With other systemic tumors; (3) Non-gastric cancer based on pathological classification; (4) Received bypass surgery or palliative surgery.

**Figure 1:**
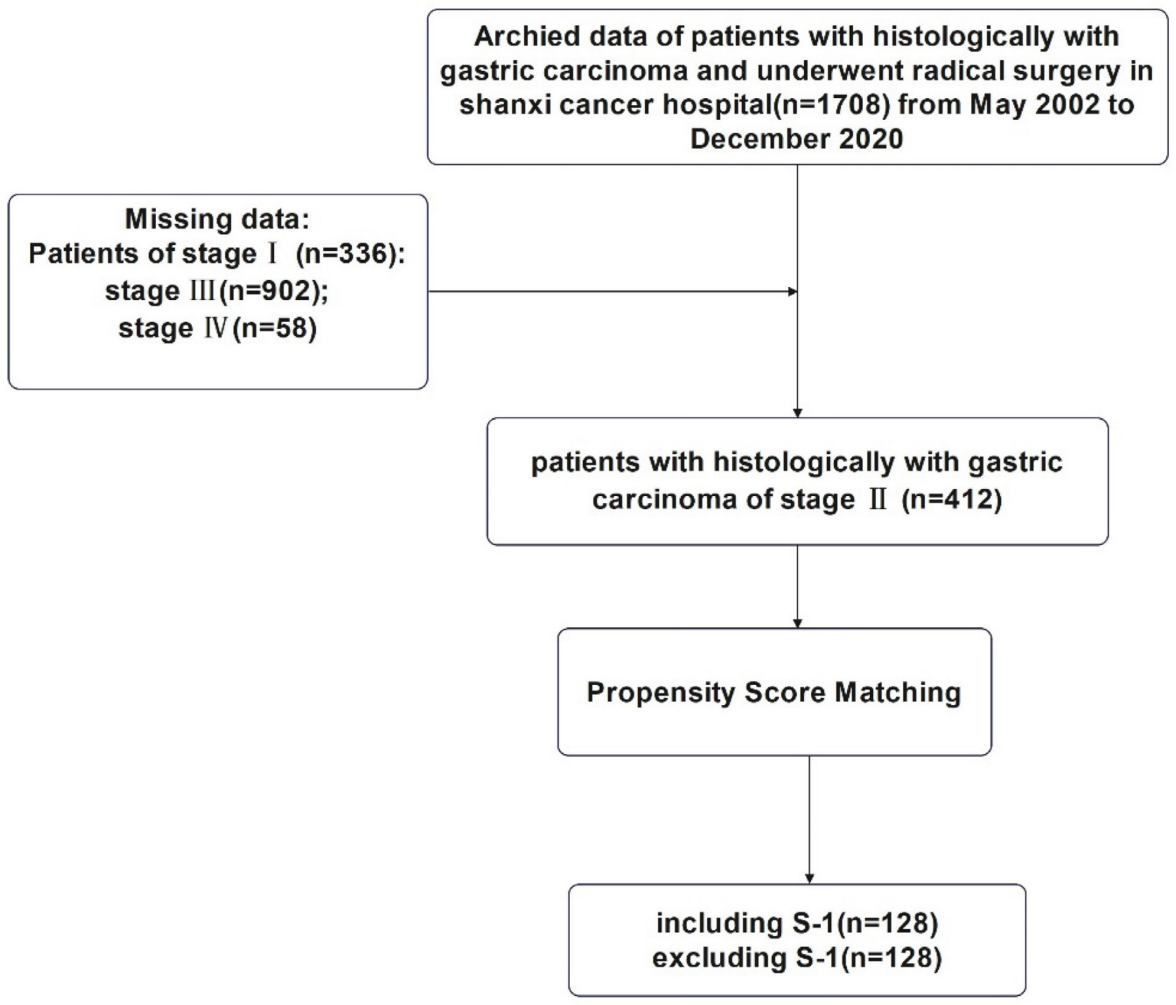
Flowchart for the enrollment of patients with stage II gastric cancer

**Figure 2:**
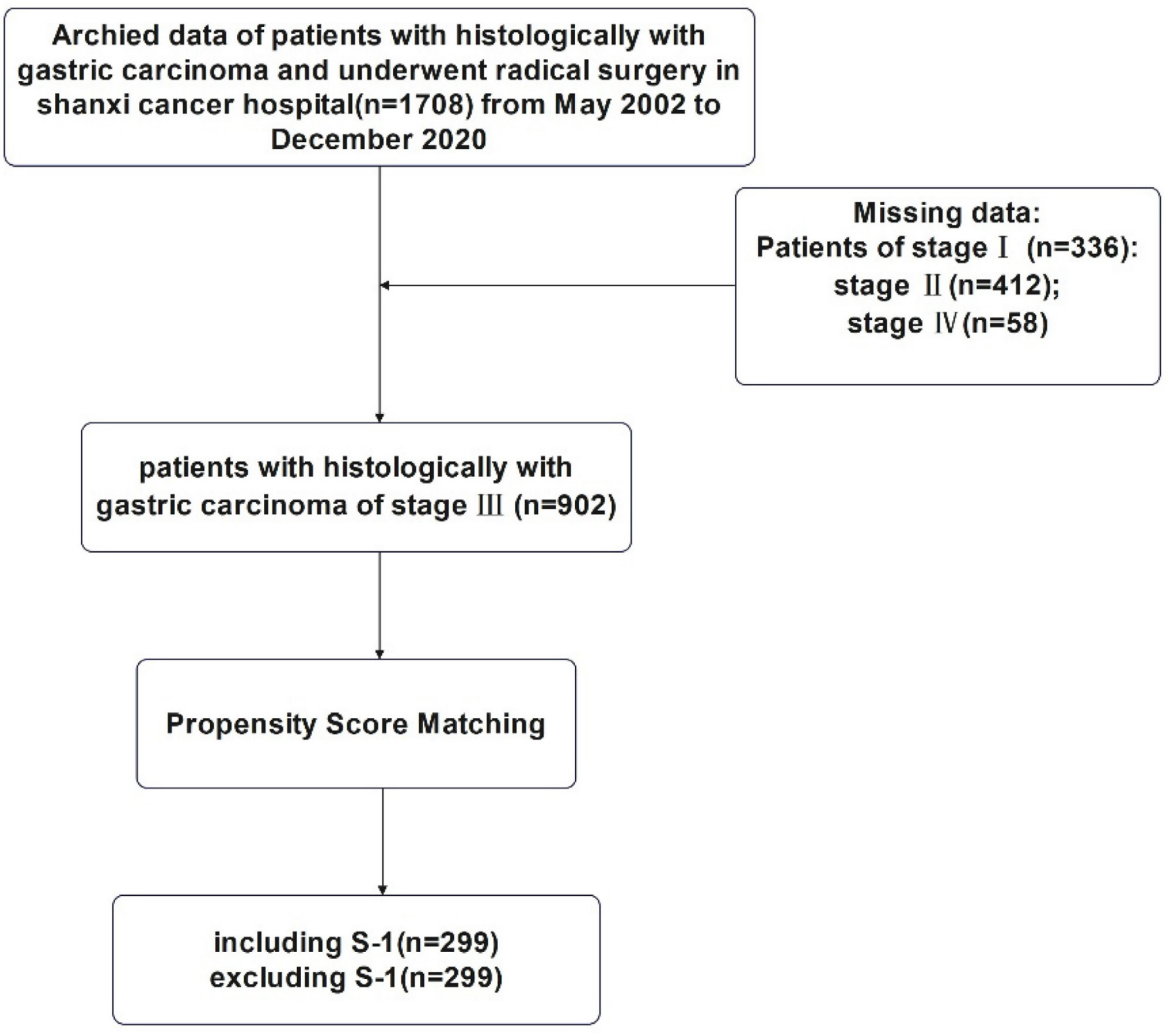
Flowchart for the enrollment of patients with stage III gastric cancer.

This study was approved by the Ethics Committee of Shanxi Cancer Hospital and was carried out in accordance with the guidelines of the Declaration of Helsinki. Patient information was anonymous and was not disclosed to the public. Since this was a retrospective study, consent of the patient was not required. The specific research content and process were shown in Figure 1 and Figure 2.

### Patient treatment

Patients included in this study has received personalized chemotherapy regimens: (1) oxaliplatin (130 mg/m2), S-1 plus oxaliplatin (SOX), S-1 (40-60 mg), twice daily for two weeks followed by a 7-day rest period;

(2) Gemelacil/Tegafur/Oataxil (S-1) (40-60 mg determined by the disease location), twice daily for two weeks followed by a 7-day rest period;

(3) S-1 + apatinib, apatinib (500 mg) administered once a day continuously and S-1 (40-60 mg) administered twice daily for two weeks followed by a 7-day rest period;

(4) Combination of Folinic acid (200 mg/m2), fluorouracil (2800 mg/m2), and oxaliplatin (85 mg/m2) (FOLFOX), folinic acid (200 mg/m2), fluorouracil (2800 mg/m2), and oxaliplatin (85 mg/m2) administered every 3 weeks;

(5) Oxaliplatin and capecitabine (also known as XELOX) were administered intravenously. Oxaliplatin (150 mg/m2) was administered on the first day of every three cycles, while capecitabine (1000 mg/m2) was taken orally twice daily for day 1 to day 14 followed by a 7-day rest period;

(6) Capecitabine (1000 mg/m2) was taken orally twice daily for two weeks followed by a 7-day rest period;

(7) Cisplatin and fluorouracil (also known as DCF), S-1 + docetaxel, cisplatin (75 mg/m2), docetaxel (75 mg/m2) on day 1 to day 5, fluorouracil (750 mg/m2) on day 1 to day 5, S-1 (40-60 mg) on day 1 to day 14, orally twice daily followed by a 7-day rest period;

(8) Oral administration of defluoruridine (1000 mg/m2) twice daily from day 1 to day 28, followed by a 14-day rest period.

Basically, chemotherapy regimens were divided into two groups: S-1 group in which chemotherapy regimens included S-1, such as SOX, single S-1, S-1 plus docetaxel, S-1 plus apatinib, or non-S-1 group in which chemotherapy regimens without S-1, such as FOLFOX, XELOX, single capecitabine.

All patients' specimens were dissected and examined to evaluate the pathological staging and the therapeutic response to neoadjuvant chemotherapy as well as the grade of tumor regression. According to Ryan criteria, Grade 0 = complete response with no residual tumor cells; Grade 1 = primary remission with scattered tumor cells; Grade 2 = moderate remission showing tumor cell aggregation with fibrosis, while Grade 3 = mild remission with substantial tumor cell retention 6. The toxicity associated with neoadjuvant chemotherapy was evaluated according to Standard 5.0, common terminology criteria for adverse events 7.

### Follow-up

Patients were followed up until December 2020, with 41.51±21.18 months follow-up for the stage II gastric cancer patients and 43.56±24.45 months for the stage III gastric cancer patients. Follow-up was conducted every 3 months in the first year after surgery and every 6 months in the 2 to 5 years followed by annually thereafter. Routine follow-up included laboratory tests, physical examinations, pelvic ultrasound, chest radiographs, magnetic resonance imaging, and computed tomography.

### Statistical analyses

Propensity score matching (PSM) analysis using 1:1 nearest neighborhood with no replacement and calipers adjusted for sample size and matching success was performed for sex, age at surgery, vascular invasion, nerve invasion, depth of tumor invasion, number of positive lymph nodes, Lauren classification, maximum tumor diameter and type of gastrectomy were used for propensity score matching (PSM) using 1:1 nearest neighborhood with no replacement and calipers adjusted for sample size and matching success. If a patient was a match, a correlation analysis of primary and secondary endpoints was performed. The primary endpoints were PFS and OS, while the secondary endpoints were tumor recurrence and metastasis, multiple metastases, and recurrence patterns.

Log-rank comparison was conducted to generate a Kaplan-Meier survival curve for each group. The categorical variable analysis was performed using appropriate tests. The P values on both sides were 0.05, which had statistical value. The date of return visit was calculated from the date of surgery to the time of last contact. OS was the time between surgery and death or the last follow-up. PFS referred to the time between surgery and the first recorded death or recurrence.

All data were analyzed using SPSS25.0 software (IBM, Armonk, NY, United States). The classification variable was expressed as percentage, and Fisher's exact test and Chi-square test were utilized. Continuous data were expressed as mean ± standard deviation, and T-test was used for analysis. Survival analysis of PFS and OS was performed using Kaplan-Meier method, which was compared with the log-rank test method. Median was used for the non-normal distribution parameters, and the analysis method was Mann-Whitney test. Subgroup analyses were performed by the Cox hazard regression model. P < 0.05 was considered statistically significant. PSM was performed with the Hansen and Bowers overall balance test. Relative multivariate imbalance L1 test was used to determine standardized mean difference < 0.25. The χ2 test was used to compare the differences in recurrence, local-regional recurrence, peritoneal metastasis, and distant metastasis between the two groups.

## Results

### PSM and subgroup analysis in TNM stage II gastric cancer patients

All cases of TNM stage II* gastric cancer patients* (n=412) with 10 variables including gender, patient's age at surgery, vascular invasion, nerve invasion, the depth of tumor invasion, the number of positive lymph nodes, Lauren classification, maximum tumor diameter, the type of gastrectomy, and surgical margin were selected for 1:1 nearest-neighborhood PSM with no replacement, and the caliper value was set to 0.1 (Table 1). Before PSM, 4 variables, age (P=0.001), depth of tumor invasion (P=0.013), type of gastrectomy (P=0.037), and Lauren classification (P=0.009) were significantly different between S-1 and non-S-1 chemotherapy regimen groups. Hence, PSM was performed in these groups, and a total of 256 eligible patients were selected, including 128 cases in the S-1 group and 128 cases in the non-S-1 group. All variables were evenly distributed between these two groups after matching (P > 0.05).

Among the 257 cases in the S-1 group, single S-1 and SOX regimens represented the major cases, 77.4% (199/257) and 16.0% (41/257), respectively. The rest cases were S-1 plus apatinib (9) and S-1 plus DCF (8). In the non-S1 group, 29.4% (125/424) received capecitabine alone, 6.4% (27/424) for Xelox, and 32.8% (140/424) for FOLFOX. The remaining 131 patients were treated with multiple chemotherapy regimen.

After PSM, the P value of Hansen & Bowers overall balance test was one and greater than 0.05. The relative multivariate imbalance L1 test showed that the value of L1 was 0.966 before PSM and was 0.961 after PSM, and there is no variable of with∣d∣>0.25 in all variables. As displayed in Figures 1 and 2, the distribution of variables between the two groups was well balanced after PSM.

**Figure 3:**
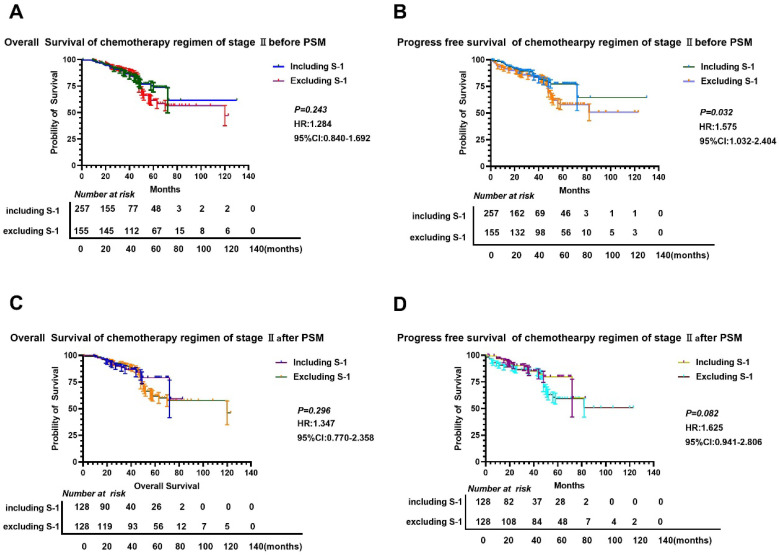
**Comparison of OS and PFS before and after PSM in stage II gastric cancer patients.** A-B: Comparison of OS (A) and PFS (B) between the two groups based on chemotherapy cycles before PSM; C-D: Comparison of OS (A) and PFS (D) between the two groups based on chemotherapy cycles after PSM.

We found that there was no significant difference in OS and PFS between S-1 and non-S-1 groups after PSM (P>0.05) (Figure 3), although the 5-year OS and PFS of patients were better in the S-1 group than in the non-S-1 group. The 1- and 3-year OS and PFS were similar between these two groups. After PSM, the median OS of S-1 group was 120 months, whereas the median OS of non-S-1 group has not yet been reached. In addition, the median PFS of patients in both groups have not yet been reached. The 1-year OS of patients in S-1 and non-S-1 groups was 97.6% and 98.4% (Log-Rank P=0.652), respectively, while the 3-year OS was 90.6% and 90.6% (Log-Rank P=1.000), respectively, indicating that the difference between these two groups was not significant. Nevertheless, there was a clear difference in 1-year PFS (96.8% VS 98.4%, Log-Rank P=0.409), 3-year PFS (80.7% VS 89.3%, Log-Rank P=0.437), and 5-year PFS (71.6% VS 61.5%, Log-Rank P=0.005) between these two groups. There was no significant difference in the recurrence (P=0.102), local-regional recurrence (P=0.062), and distant metastases (P=0.328) between these two groups. As for the recurrence patterns, the proportion of local-regional metastasis and distant metastasis were 3.13% (4/128) and 8.59% (11/128), respectively, in the S-1 group, while they were 4.57% (7/128) and 8.59% (11/128), respectively, in the non-S-1 group.

**Figure 4:**
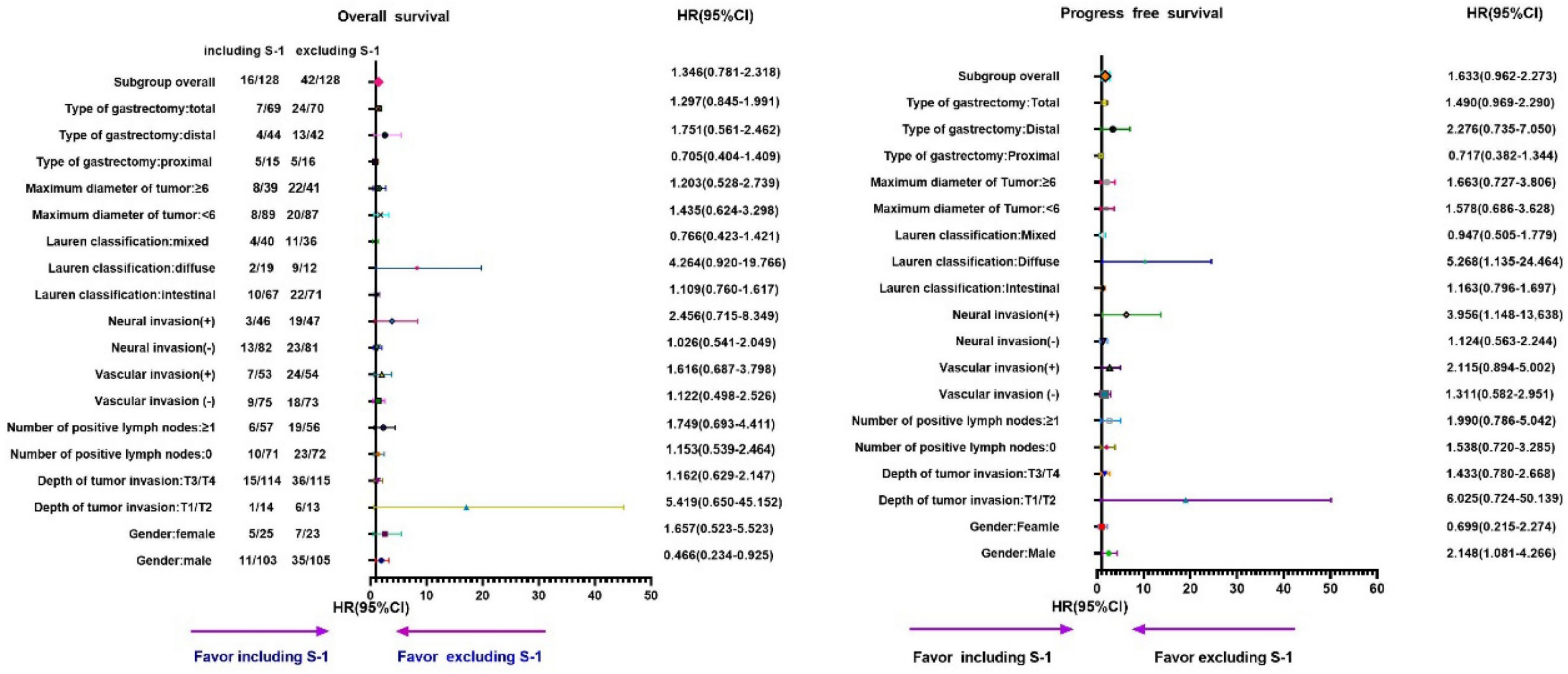
**Subgroup analyses of OS and PFS based on different chemotherapy regimens in stage II gastric cancer patients.** A: OS; B: PFS.

Furthermore, subgroup analyses showed that the OS and PFS in the S-1 group were worse than that in the non-S-1 group (Table 2, 3 and Figure 3, 4). In addition, in the subgroup of male with neural invasion (+), the PFS was markedly worse in the S-1 group than in the non-S-1 in PFS, which was significantly associated with the deterioration of PFS (P<0.05). Compared with the non-S-1 group, the mortality hazard of the negative and positive expression of vascular invasion in the S-1 group was increased by 12.2% and 61.6%, respectively, (P for interaction=0.021), while that of neural invasion was increased by 2.6% and 145.6%, respectively, (P for interaction=0.048). The disparity of maximum diameter of tumor <6cm and ≥6cm in the S-1 group was significant (P for interaction <0.001).

Furthermore, compared to the non-S-1 group, the disease progression hazard of the negative and positive expression of vascular invasion in the S-1 group was increased by 28.4% and 48.2%, respectively, (P for interaction =0.008), while that of neural invasion in the S-1 group was increased by 2.1% and 136.8%, respectively, (P for interaction =0.022). The maximal diameter of tumor <6cm and ≥6cm in disease progression hazard of the S-1 group was increased by 26.2% and 47.1%, respectively, (P for interaction <0.001).

### PSM and subgroup analysis of patients with TNM stage III gastric cancer

1:1 nearest-neighborhood PSM with no replacement was performed on all cases with TNM stage III (n=902), and the caliper value was set to 0.05 (Table 2). At the end, a total of 598 patients were eligible, including 299 cases in S-1 group and 299 cases in non-S-1 group. Before PSM, there was a significant difference in age (P<0.001) and the depth of tumor invasion (P<0.001) between patients in the S-1 and non-S-1 groups; however, after matching, these variables were evenly distributed between the two groups (P > 0.05).

In the S-1 group, the proportions of single S-1 and SOX regimens were 80.5% (383/476) and 10.1% (49/476), respectively. For the remaining cases, 10 received S-1 plus apatinib; 6 received S-1+DCF; 20 received SOX+FOLFOX, and 8 received S-1+FOLFOX. In the non-S-1 group, the proportion of capecitabine alone was 29.4% (125/424), while 6.4% (27/424) were treated with Xelox, and 32.8% (140/424) received FOLFOX. The remaining 131 patients received a combination chemotherapy of multiple chemotherapy regimens.

After PSM, the P value of Hansen & Bowers overall balance test was one and greater than 0.05. The relative multivariate imbalance L1 test showed that the value of L1 was before PSM and 0.906 after PSM. There was no variable of with∣d∣>0.25 in all variables. As shown in Figures 1 and 2, the distribution of variables was well balanced between the two groups after PSM.

**Figure 5:**
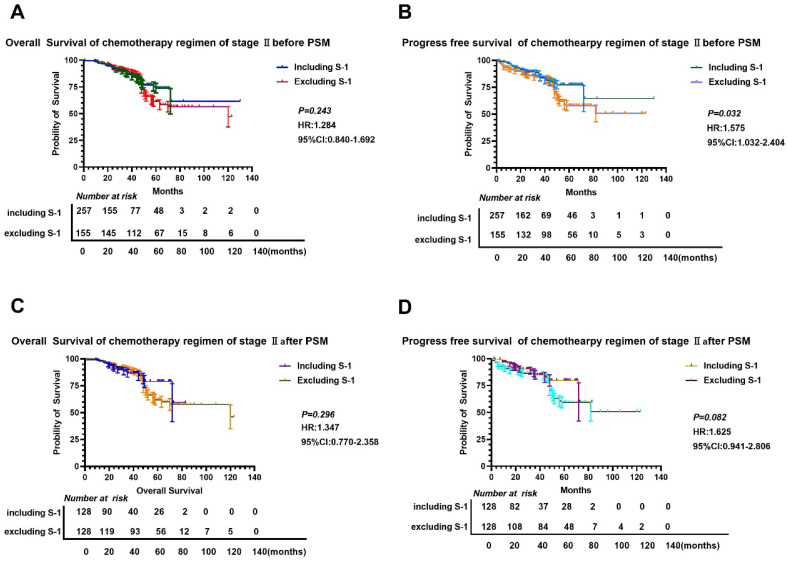
** Comparison of OS and PFS before and after PSM in stage III gastric cancer patients.** A-B: Comparison of OS (A) and PFS (B) between the two groups based on chemotherapy cycles before PSM; C: Comparison of OS (C) and PFS (E) between the two groups based on chemotherapy cycles after PSM.

There was a clear difference in OS between the S-1 and non-S-1 groups regardless of PSM (P < 0.05), as patients in the S-1 group showed a better OS compared to patients in the non-S-1 group (Figure 5). Similarly, the 1- and 3-year PFS of patients in the S-1 group was better than that of patients in the non-S-1 group. However, the 5-year PFS as well as the 1-, 3-, and 5-year OS were not significantly different between the two groups. Before PSM, the median OS of the S-1 and non-S-1 groups was 37 and 48 months, respectively, while the PFS of these two groups was 35 and 44 months, respectively. After PSM, the median OS of the S-1 and non-S-1 groups was 37 and 48 months, respectively. Further comparison between these two groups showed that the 1-year (36.4% VS 12.0%, Log-Rank P=0.050), 3-year (39.6% VS 21.9%, Log-Rank P=0.095), and 5-year OS (42.2% VS 25.7%, Log-Rank P=0.264) was not significantly different. As for PFS, the median PFS of the S-1 and non-S-1 groups was 35 and 44 months, respectively. Importantly, there was a significant difference between these two groups in the 1-year (26.4% VS 17.2%, Log-Rank P=0.013) and 3-year PFS (37.8% VS 25.0%, Log-Rank P=0.003). Moreover, the occurrence of recurrence, local metastases, distant metastases, and multiple metastases was attenuated in the S-1 group. Specifically, the recurrence rates were 23.75% (71/299) and 39.46% (118/299), respectively, for the S-1 and non-S-1 groups, and there was a significant difference in recurrence (P<0.001), local-regional recurrence (P=0.002), and distant metastases (P=0.011) between these two groups. For the recurrence patterns, the proportion of local-regional metastasis, peritoneal metastasis and distant metastasis were 39.44% (25/71), 11.27% (8/71), and 53.52% (38/71), respectively, in the S-1 group; however, they were 42.37% (50/118), 5.93% (7/118), and 51.69% (61/118), respectively, in the non-S-1 group.

**Figure 6:**
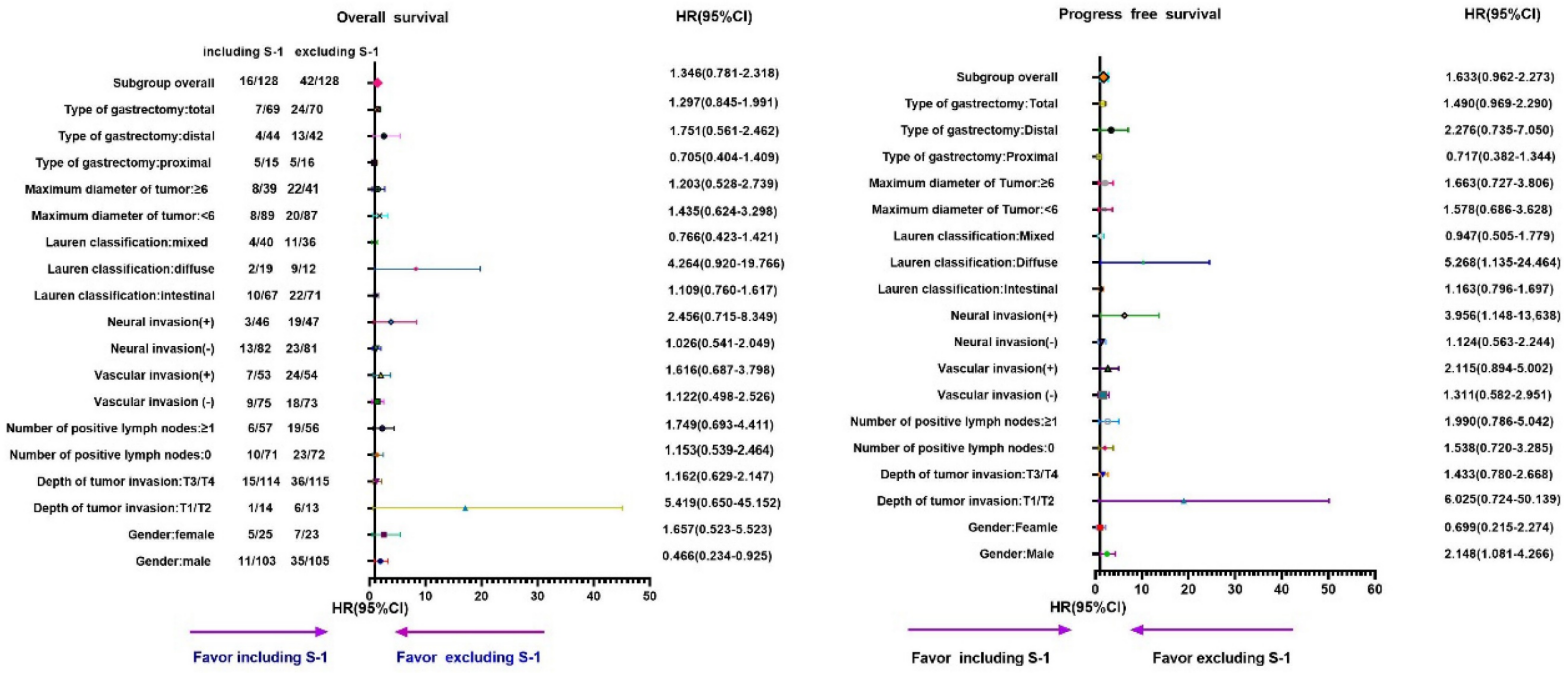
**Subgroup analyses of OS and PFS for different chemotherapy regimens in stage III gastric cancer patients.** A-B: Subgroup analyses of OS (A) and PFS (B) based on chemotherapy cycles.

In addition, we found that the mortality hazard was increased by 27.2% in the S-1 group compared to the non-S-1 group (P=0.023), as determined by the subgroup analyses of OS (Table 5 and Figure 5) although the difference in PFS was not statistically significant (P=0.268) in subgroup analyses (Table 6 and Figure 6). In the subgroup analysis on sex, T stage, the number of positive lymph nodes ≤2, the number of positive lymph nodes ≥3, positive vascular invasion, positive neural invasion, the maximum diameter of tumor ≥6 cm, diffuse type, and total gastrectomy, patients in the S-1 group showed significantly worse OS than those in the non-S-1 group (P<0.05). Compared to the S-1 group, the number of positive lymph nodes ≤2 and ≥3 in disease progression hazard were reduced by 20.1% and increased by 0.7%, respectively, in the non-S-1 group (P for interaction=0.003).

## Discussion

Chemotherapy regimen including S-1 has become the first-line and second-line regimen for adjuvant chemotherapy for gastric cancer. In addition, many clinical trials have tested the safety and tolerance of S-1 for gastric cancer patients with different TNM stages. For example, one study has investigated the efficacy and safety of including S-1 chemotherapy in SOX for Stage III gastric cancer patients after radical resection and found a beneficial effect 8. Another study focused on the efficacy and safety of SOX as neoadjuvant chemotherapy for locally advanced gastric cancer and esophagogastric junction cancer and also showed better treatment outcome 9. Similarly, a study has compared the therapeutic effects of S-1 to S-1 plus docetaxel on 3-year OS and recurrence-free survival (RFS) in patients with stage III gastric cancer and found that S-1 plus docetaxel significantly improved the OS and RFS 10. In another study of evaluating the efficacy and side effects of low-dose oxaliplatin combined with pegylated liposomal doxorubicin and S-1 as the first-line treatment for advanced gastric cancer, it was found that the combination of low-dose oxaliplatin with S-1 and pegylated liposomal doxorubicin improved OS and PFS as well as presented lower incidence of neurotoxicity compared to the standard SOX 11. Furthermore, one study investigated the efficacy and safety of intraperitoneal and systemic paclitaxel chemotherapy combined with apatinib and S-1 in patients with positive exfoliative cytology of gastric cancer, in which all patients with negative cytology underwent R0 resection with a median follow-up of 11.4 months, and the results showed that the median OS was 15.5 months, and 80.55% of patients had 1-year OS, while the median PFS was 14.4 months with 75.00% patients had 1-year PFS 12, demonstrating that this combination therapy could improve the negative rate of exfoliative cytology. A similar study evaluated the efficacy and safety of S-1 cisplatin combined with cetuximab as the first-line chemotherapy for Japanese patients with advanced gastric cancer and determined that 1 out of 40 patients (2.5%) had a complete response, while 15 patients (37.5%) had a partial response. The overall response rate was 40.0%, and the median PFS was 5.6 months 13.

Although numerous studies have indicated the increased efficacy of including S-1, the effect of S-1 in gastric cancer remains to be determined. In this study, we demonstrated that including S-1 did not show superiority over excluding S-1 in the prognosis of stage II and III gastric cancer patients, but instead, it increased the risk of mortality of stage Ⅲ gastric cancer patients and the chance of recurrence of stage II and III cancer. Nevertheless, compared with 5 -fluorouracil, S-1 showed several advantages. First, the higher drug concertation could result in higher anticancer activity. Second, the combination therapy could lead to less drug toxicity with a convenience of oral administration. However, including S-1 in the regimen could still cause side effects, such as lipsotrichia, reduced immunity, nausea, vomiting, and diarrhea, as well as escalated the psychological symptoms such as anxiety. Thus, selecting other regimen plus S-1 or other regimens for advanced gastric cancer is urgently needed. Unlike previous research, we first classified including S-1 as an entirety compared to excluding S-1 in terms of prognosis and the results are totally contrary to former understanding about the role of S-1 in treatment of gastric cancer.

There were several limitations in the current study. First, as a retrospective analysis conducted at a single center, this study was subjected to possible selection bias despite the use of PSM to reduce bias. The use of PSM was intended to mimic randomized controlled trials. Second, the regimens and indications for chemotherapy were not standardized; therefore, the effects of different chemotherapy regimens were not analyzed. Nonetheless, the interactive effect between chemotherapy regimens and vascular invasion, neural invasion, and the maximum diameter of the tumor on OS and PFS were determined for the first time.

## Figures and Tables

**Table 1:** The clinicopathological features of stage II gastric cancer patients before and after PSM of chemotherapy regimen

Variables	Before PSM		P	After PSM		P
	Including S-1 (n=257)	Excluding S-1 (n=155)		Including S-1 (n=128)	Excluding S-1 (n=128)	
Gender			0.771			0.749
Male	211	129		103	105	
Female	46	26		25	18	
Age(year)	60.06±10.50	56.98 ± 10.05	<0.001	58.66±8.87	58.90±8.99	0.739
Depth of tumor invasion			0.013			0.577
T1	6	7		4	3	
T2	17	12		10	10	
T3	190	84		83	85	
T4	44	52		31	35	
Number of positive lymph nodes			0.514			0.860
0	143	93		71	72	
1-2	106	54		52	52	
3-6	4	5		2	2	
≥7	4	3		3	2	
Type of gastrectomy			0.037			0.959
Proximal	19	19		11	12	
Distal	79	55		35	36	
Total	159	81		63	61	
Vascular invasion			0.512			0.801
Negative	156	89		75	73	
Positive	101	66		53	55	
Neural invasion			0.845			0.897
Negative	160	95		82	81	
Positive	97	60		46	47	
Lauren classification			0.009			0.577
Intestinal	112	91		15	16	
Diffuse	61	24		44	42	
Mixed	84	40		69	70	
Maximum diameter of tumor (cm)			0.301			0.788
<6	188	106		70	75	
≥6	69	49		27	22	
Surgical margin			0.139			0.562
Negative	250	154		126	127	
Positive	7	1		2	1	

**Table 2:** Subgroups analysis of OS by cox regression of chemotherapy regimen for stage II gastric cancer patients

Variables	Event	Total	HR	95% CI	P	P for interaction
Gender						0.586
Male	46	208	0.466	0.234-0.925	0.146	
Female	12	48	1.657	0.523-5.523	0.391	
Depth of tumor invasion						0.739
T1/T2	7	27	5.419	0.650-45.152	0.188	
T3/T4	51	229	1.162	0.629-2.147	0.632	
Number of positive lymph nodes						0.231
0	33	143	1.153	0.539-2.464	0.714	
≥1	25	113	1.749	0.693-4.411	0.237	
Vascular invasion						0.729
Negative	27	148	1.122	0.498-2.526	0.782	
Positive	31	108	1.616	0.687-3.798	0.271	
Neural invasion						0.048
Negative	36	163	1.026	0.541-2.049	0.941	
Positive	22	93	2.456	0.715-8.349	0.154	
Lauren classification						0.700
Intestinal	32	138	1.109	0.760-1.617	0.592	
Diffuse	11	42	4.264	0.920-19.766	0.064	
Mixed	15	76	0.766	0.423-1.421	0.411	
Maximum diameter of tumor (cm)						<0.001
<6	28	176	1.435	0.624-3.298	0.395	
≥6	30	80	1.203	0.528-2.739	0.660	
Type of gastrectomy						0.206
Proximal	31	139	0.755	0.404-1.409	0.377	
Distal	17	86	1.751	0.561-5.462	0.355	
Total	10	31	1.297	0.845-1.991	0.234	

**Table 3:** Subgroups analysis of PFS by cox regression of chemotherapy regimen for stage II gastric cancer patients

Variables	Event	Total	HR	95% CI	P	P for interaction
Gender						0.628
Male	55	208	1.997	1.078-3.699	0.028	
Female	17	48	0.488	0.183-1.302	0.152	
Depth of tumor invasion						0.591
T1/T2	9	27	3.609	0.748-17.415	0.110	
T3/T4	63	229	1.234	0.721-2.111	0.444	
Number of positive lymph nodes						0.230
0	39	143	1.450	0.721-2.919	0.297	
≥1	33	113	1.382	0.663-2.883	0.388	
Vascular invasion						0.008
Negative	32	148	1.284	0.613-2.690	0.508	
Positive	40	108	1.482	0.738-2.979	0.269	
Neural invasion						0.022
Negative	44	163	1.021	0.548-1.901	0.949	
Positive	28	93	2.368	0.941-5.960	0.067	
Lauren classification						0.210
Intestinal	37	138	1.166	0.589-2.309	0.660	
Diffuse	14	42	4.246	1.181-15.265	0.027	
Mixed	21	76	0.783	0.283-2.169	0.638	
Maximum diameter of tumor (cm)						<0.001
<6	38	176	1.262	0.624-3.298	0.507	
≥6	34	80	1.471	0.528-2.739	0.317	
Type of gastrectomy						0.134
Proximal	12	139	0.398	0.124-1.281	0.122	
Distal	31	86	1.962	0.760-5.065	0.163	
Total	37	31	1.793	0.854-3.766	0.123	

**Table 4:** The clinicopathological features of stage III gastric cancer patients before and after PSM of chemotherapy regimen

Variables	Before PSM		P	After PSM		P
	Including S-1(n=476)	Excluding S-1(n=426)		Including S-1(n=299)	Excluding S-1(n=299)	
Gender			0.172			0.839
Male	380	324		237	239	
Female	96	102		62	60	
Age(year)	60.33±9.68	57.26±10.29	<0.001	59.36±9.79	58.20±9.78	0.139
Depth of tumor invasion			<0.001			0.535
T2	0	2		0	0	
T3	161	63		55	61	
T4	315	361		244	238	
Number of positive lymph nodes			0.107			0.544
0	3	1		0	1	
1-2	66	84		56	52	
3-6	121	102		78	73	
≥7	286	239		165	173	
Type of gastrectomy			0.207			0.785
Proximal	25	27		21	17	
Distal	122	121		77	79	
Total	329	278		201	203	
Vascular invasion			0.831			0.548
Negative	118	103		77	71	
Positive	358	323		222	228	
Neural invasion			0.831			0.548
Negative	163	143		107	100	
Positive	313	283		192	199	
Lauren classification			0.176			0.708
Intestinal	98	79		63	62	
Diffuse	225	243		155	163	
Mixed	153	104		81	74	
Maximum diameter of tumor (cm)			0.962			1.000
<6	241	215		148	148	
≥7	235	211		151	151	
Surgical margin			0.268			0.874
Negative	446	391		278	277	
Positive	30	35		21	22	

**Table 5:** Subgroups analysis of OS by Cox regression of chemotherapy regimen of stage Ⅲ gastric cancer patients

Variables	Event	Total	HR	95% CI	P	P for interaction
Gender						0.637
Male	267	476	0.715	0.561-0.911	0.007	
Female	75	122	0.722	0.453-1.151	0.171	
Depth of tumor invasion						0.113
T3	39	116	1.022	0.514-2.032	0.951	
T4	303	482	0.706	0.562-0.886	0.003	
Number of positive lymph nodes						0.476
≤2	40	109	0.492	0.261-0.928	0.029	
≥3	302	489	0.771	0.613-0.969	0.026	
Vascular invasion						0.254
Negative	67	148	0.767	0.473-1.244	0.282	
Positive	275	450	0.708	0.557-0.990	0.005	
Neural invasion						0.598
Negative	97	207	0.784	0.523-1.176	0.239	
Positive	245	391	0.699	0.543-0.901	0.006	
Lauren classification						0.363
Intestinal	55	125	0.727	0.555-0.952	0.020	
Diffuse	216	318	0.710	0.541-0.931	0.013	
Mixed	71	155	0.939	0.742-1.189	0.603	
Maximum diameter of tumor (cm)						0.584
<6	150	296	0.755	0.546-1.044	0.089	
≥6	196	302	0.704	0.528-0.940	0.017	
Type of gastrectomy						0.288
Proximal	16	38	0.852	0.519-1.399	0.527	
Distal	78	156	0.789	0.504-1.235	0.300	
Total	248	404	0.820	0.722-0.931	0.002	

**Table 6:** Subgroups analysis of PFS by Cox regression of chemotherapy regimen of stage Ⅲ gastric cancer patients

Variables	Event	Total	HR	95% CI	P	P for interaction
Gender						0.070
Male	299	476	0.946	0.751-1.191	0.637	
Female	88	122	1.020	0.663-1.568	0.929	
Depth of tumor invasion						0.945
T3	59	116	1.148	0.655-2.012	0.631	
T4	328	482	0.943	0.757-1.175	0.600	
Number of positive lymph nodes						0.003
≤2	48	109	0.799	0.445-1.435	0.453	
≥3	339	489	1.007	0.811-1.250	0.953	
Vascular invasion						0.508
Negative	78	148	1.205	0.764-1.889	0.423	
Positive	309	450	0.909	0.724-1.140	0.408	
Neural invasion						0.144
Negative	115	207	1.010	0.695-1.468	0.958	
Positive	272	391	0.956	0.751-1.217	0.713	
Lauren classification						0.737
Intestinal	68	125	0.907	0.559-1.471	0.693	
Diffuse	239	318	0.866	0.644-1.122	0.275	
Mixed	80	155	1.254	0.832-1.968	0.325	
Maximum diameter of tumor (cm)						0.114
<6	173	296	1.069	0.788-1.451	0.667	
≥6	214	302	0.886	0.675-1.164	0.386	
Type of gastrectomy						0.576
Proximal	23	38	2.036	0.869-4.773	0.102	
Distal	90	156	0.870	0.572-1.323	0.512	
Total	274	404	0.923	0.724-1.177	0.519	

## Abbreviations

PSM: propensity score matching
OS: overall survival
PFS: progress free survival
